# Food for thought—The link between *Clostridioides difficile* metabolism and pathogenesis

**DOI:** 10.1371/journal.ppat.1011034

**Published:** 2023-01-05

**Authors:** Andrew Marshall, John W. McGrath, Robert Graham, Geoff McMullan

**Affiliations:** School of Biological Sciences, Queen’s University Belfast, Belfast, Northern Ireland, United Kingdom; Geisel School of Medicine at Dartmouth, UNITED STATES

## Abstract

*Clostridioides difficile* (*C*. *difficile*) is an opportunistic pathogen that leads to antibiotic-associated diarrhoea and is a leading cause of morbidity and mortality worldwide. Antibiotic usage is the main risk factor leading to *C*. *difficile* infection (CDI), as a dysbiotic gut environment allows colonisation and eventual pathology manifested by toxin production. Although colonisation resistance is mediated by the action of secondary bile acids inhibiting vegetative outgrowth, nutrient competition also plays a role in preventing CDI as the gut microbiota compete for nutrient niches inhibiting *C*. *difficile* growth. *C*. *difficile* is able to metabolise carbon dioxide, the amino acids proline, hydroxyproline, and ornithine, the cell membrane constituent ethanolamine, and the carbohydrates trehalose, cellobiose, sorbitol, and mucin degradation products as carbon and energy sources through multiple pathways. Zinc sequestration by the host response mediates metabolic adaptation of *C*. *difficile* by perhaps signalling an inflamed gut allowing it to acquire abundant nutrients. Persistence within the gut environment is also mediated by the by-products of metabolism through the production of *p*-cresol, which inhibit gut commensal species growth promoting dysbiosis. This review aims to explore and describe the various metabolic pathways of *C*. *difficile*, which facilitate its survival and pathogenesis within the colonised host gut.

## Author summary

*Clostridioides difficile* (*C*. *difficile*) infection (CDI) is a global health threat being the leading cause of antibiotic-associated diarrhoea. CDI is mediated by the toxins TcdA and TcdB, which act to damage the gut epithelium leading to a breakdown in the gut barrier integrity. In addition, CDI possesses a high recurrence rate, owing to the ability of *C*. *difficile* to produce highly resilient endospores allowing it to persist following antibiotic treatment regimes. The ability of a pathogen to successfully colonise the gut relies on its ability to acquire and metabolise nutrients from its environment. *C*. *difficile* is proposed to act as a bacterial generalist, capitalising on the broad range of available nutrients present within a dysbiotic gut environment. This review describes the metabolic capabilities of *C*. *difficile* within the current literature to provide an overview of the genetic and molecular basis in which its metabolism operates following gut colonisation and how this contributes towards pathogenicity.

## Introduction

Colonisation resistance is the ability of the gut microbial community to inhibit and outcompete pathogenic organisms preventing them from colonising the gut environment [1]. *Clostridioides* (formerly *Clostridium*) *difficile* is a spore forming, gram-positive bacterium that is the major cause of antibiotic-associated diarrhoea worldwide and whose pathogenic lifestyle relies on an ability to produce the toxins TcdA and TcdB [2]. Gut microbiota-mediated colonisation resistance against *C*. *difficile* occurs via the conversion of primary bile acids to secondary bile acids, thus preventing the germination of its spores [3]. Additionally, consumption of Stickland metabolism substrates by primary bile acid metabolising species within the gut also contributes to the outcompeting of *C*. *difficile* in vivo [4]. However, when the normal gut microbiota is disrupted through, for example, antibiotic therapy, the resultant perturbation of the structure and function of the gut microbiota leads to a dysbiotic state that favours *C*. *difficile* colonisation [1].

The ability of the gut microbiota to occupy nutrient niches and restrict the use of nutrients therein is thought to play a key role in preventing *C*. *difficile* colonisation. Wilson and Sheagren (1983), for example, reported that precolonisation by a nontoxigenic strain of *C*. *difficile* in a mouse model protected against colonisation by a toxigenic strain [5] while the colonic microbiota of a mouse was found to outcompete *C*. *difficile* by depleting carbohydrates in a gut model environment [6]. Similarly, colonisation of hamster models with nontoxigenic *C*. *difficile* prevented colonisation by toxigenic strains, although surprisingly, this protection could not be provided by other *Clostridium* species [7–9]. *Paraclostridium bifermentans* has also been shown to prevent *C*. *difficile* colonisation in vivo by consuming glycine, a cogerminant for spores [3,10]. Taken together, it is reasonable to hypothesise that when an ecological niche required by *C*. *difficile* is occupied, then the pathogen is outcompeted and unable to colonise.

*C*. *difficile* possesses a large and mosaic genome, capable of metabolising a diverse range of nutrients for growth [11]. Carbohydrates and amino acids, which we have already indicated are of importance in colonisation resistance towards *C*. *difficile*, are enriched in the gut metabolome following antibiotic disruption of the colonic microbiota [12]. This allows *C*. *difficile* to capture these “free” nutrients vital for its outgrowth post-spore germination using a specific subset of metabolic pathways, regardless of the gut community structure in a post-antibiotic environment [13–15]. Virulence of *C*. *difficile* also has an intimate relationship with nutrient availability as, upon nutrient limitation in the gut environment, *C*. *difficile* responds by producing toxins that liberate host-derived nutrients through cytotoxic activity and creation of a proinflammatory environment [16]. The molecular basis of this has begun to be dissected, and it is known that the global nutrient regulators CcpA (glucose), CodY (branch chained amino acids and GTP), PrdR (proline), and Rex (NADH/NAD^+^ ratio) inhibit toxin expression when their cognate nutrient is present in excess [17–21]. *C*. *difficile’s* production of toxins can then be thought of as a means to maintain a favourable nutrient environment to sustain growth during infection. The purpose of this review is to highlight recent advances in our understanding of how the interplay between *C*. *difficile*, its host and the gut microbiota—with respect to the availability and metabolism of certain nutrients—is implicated in the pathogenesis of this important gut pathobiont (Table 1).

**Table 1:** Nutrients of interest utilised by *C*. *difficile* during gut colonisation.

Nutrient	Metabolic pathway	Metabolic end product(s)	Genes	Reference
Proline	Stickland metabolism	5-aminovalerate + NAD^+^	*prd* operon	[20,22]
Glycine	Stickland metabolism	Acetate + NAD^+^ + ATP	*grd* operon	[20,22]
Hydroxyproline	Stickland metabolism	5-aminovalarte + NAD^+^	*hypD* and *prd* operon	[23]
Ornithine	Stickland metabolism	NADH + Ammonia + Acetyl-CoA + Alanine	*orr*, *oraSE*, *ord*, and *ortAB*	[24]
CO_2_	Wood–Ljungdahl pathway	Acetyl-CoA + Acetate	*acsA*, *cooC*, *fhs*, *fchA*, *folD*, *metV*, *metF*, *gcvL*, *acsF*, *acsD*, a*csC*, *acsE*, *acsB*, *gcvH*, *acsV*	[25]
Ethanolamine	Central carbon metabolism	Ethanol + Acetyl-CoA + Acetate	*eut* gene cluster	[26]
Sorbitol	Carbohydrate metabolism	Fructose-6-phosphate + NADH	*srlR*, *srlM*, *srlAebB*, and *srlD*	[27]
Trehalose	Carbohydrate metabolism	Glucose + Glucose-6-phosphate	*treA*, *treR* and/or *treA2*, *ptsT*, *treX*, and *treR2*	[28,29]
Cellobiose	Carbohydrate metabolism	Glucose + Glucose-6-phoshate	*celR*, *celA*, *celB*, *celF*, and *celC*	[30]
Sialic acid (5-acetylneuraminate)	Carbohydrate metabolism	Pyruvate, Acetate + Fructose-6-phosphate	*nanE*, *nanA*, *nanT*, and *nanK*	[31]
Mannose	Carbohydrate metabolism	Fructose-6-phosphate	CD630_24910, CD630_02860–02890	[32]
Zinc	N/A	N/A	*zupT*	[33]
Tyrosine	Stickland metabolism	*p*-cresol	*hpdBCA* operon	[34,35]

### The importance of Stickland metabolism for gut colonisation

Stickland metabolism is of great importance in *Clostridium* species, and *C*. *difficile* is no exception. Stickland metabolism involves the coupled oxidation and reduction of two amino acids, where one acts as an electron donor and the other as an electron acceptor, facilitating the production of ATP by substrate-level phosphorylation and maintaining the NADH/NAD^+^ pool [36]. The most efficient electron donor include the amino acids alanine, valine, leucine, and isoleucine [37–39], and the most efficient electron acceptors include, proline, glycine, and leucine [22,40,41].

Early studies defining growth requirements in *C*. *difficile* described six amino acids as essential for its growth including, leucine, isoleucine, valine, tryptophan, proline, and glycine [42]. Proline and glycine are thought as the most important Stickland electron acceptors in *C*. *difficile* with D-proline being reduced to 5-aminovalerate by proline reductase (PR), and glycine reduced to acetyl phosphate by glycine reductase (GR) [20], with both enzymes requiring selenium for maximal activity [22]. The proline reductase (*prd*) operon is induced by the presence of its substrate through the regulatory protein, PrdR, which also leads to the repression of the glycine reductase (*grd*) operon [20]. While proline reduction in *Clostridium sporogenes* has been shown to be coupled to the production of a proton motive force allowing ATP generation, this has yet to be characterised in *C*. *difficile* [43]. Proline is one of the first amino acids to be rapidly consumed by *C*. *difficile*, accompanied by its reduction to 5-aminovalerate [25,44,45], where the repression of the *grd* operon in excess proline is thought to shuttle all available selenium towards PR as this is the main means of NAD^+^ regeneration in *C*. *difficile* metabolism [46]. *C*. *difficile* is then thought to manage Stickland metabolism in a hierarchical manner, preferentially utilising specific amino acids as substrates to maximise energy intake during growth.

In vivo mice model studies have also demonstrated the importance of proline for *C*. *difficile* colonisation with free proline shown to increase in a dysbiotic state [47]. In humanised microbiome mice infected with a *prdB* mutant [47], decreased colonisation was observed, while mice colonised with either *Clostridium leptum*, *Clostridium scindens*, or *Clostridium hiranonis* depleted proline, protecting against CDI [4]. Further, in vivo transcriptomics have demonstrated that toxin-mediated inflammation alters the gut nutrient environment to one that favours *C*. *difficile* metabolic needs, specifically amino acid metabolism through the release of proline and hydroxyproline from collagen (see below) [16]. Unsurprisingly then, utilisation of proline by *C*. *difficile* has been shown to be of importance for successful colonisation of the gut once dysbiosis occurs [47].

Hydroxyproline, a host-modified form of proline and a major constituent of collagen, can be converted to L-proline by the glycyl radical enzyme, 4-hydroxyproline reductase (*hypD*) and a pyrroline-5-carboxylate reductase (*proC*), with the former essential for hydroxyproline-dependent growth in *C*. *difficile* [23,48]. L-proline is then racemised by a proline racemase (*prdF)*, with the resultant D-proline being the substrate for PR [49]. Hydroxyproline could be generated through *C*. *difficile* toxin activity, which induces the production of host matrix metalloproteases in vivo to degrade collagen, liberating hydroxyproline (and proline) to be metabolised [16,50]. Toxin activity has also been shown to reduce and reorganise the amount of collagen found around cells in vitro, potentially allowing greater ease of access to Stickland metabolism substrates [16]. Further, the effects of toxin activity were demonstrated to suppress the Bacteroidaceae family, whose members were shown to be enriched in homologues of the *hypD* gene and thus may exclude a competitor that could compete with *C*. *difficile* for amino acids [16,51]. The degradation of collagen during toxin-mediated inflammation may then be an important source of metabolic substrates such as proline and hydroxyproline to fuel *C*. *difficile* Stickland metabolism and maintain redox balance. Of note, *C*. *difficile* strains show differing abilities to metabolise hydroxyproline with R20291 and VPI 10463 strains having *hypD* expression induced 10-fold in its presence, whereas the 630 strain showed no expressional change [23]. As a result, the efficiency of hydroxyproline utilisation by *C*. *difficile* in the gut may differ depending on the colonising strain, and only a subset of strains may benefit by its release from degraded collagen.

Supporting this hypothesis, an inability to metabolise hydroxyproline by a *hypD C*. *difficile* mutant in a mouse model of CDI led to decreased fitness of the pathogen, less toxin production, and an increased abundance of *Lachnospiraceae*, a gut commensal species antagonistic to *C*. *difficile* [23]. Additionally, in antibiotic-treated mice in vivo metabolomics studies demonstrated an increased abundance of both proline and hydroxyproline, where upon *C*. *difficile* colonisation, both were shown to decrease with a concomitant increase of the metabolic by-product, 5-aminovalerate [4,10,13,16,52]. Significant increases in 5-aminovalerate have also been observed in patients with CDI, suggesting the active metabolism of proline and/or hydroxyproline by *C*. *difficile* following colonisation of the human gut [4]. Altogether, proline and hydroxyproline can be thought of as important amino acids that facilitate colonisation success, whereby toxin-mediated inflammation could be a main driver for their production to be utilised in *C*. *difficile* Stickland metabolism.

### The role of the Wood–Ljungdahl pathway during nutrient limitation

In acetogenic bacteria, the Wood–Ljungdahl pathway (WLP) functions to reduce CO_2_ to acetyl-CoA and acetate, acting as an electron sink to dispose of reducing equivalents and regenerate NAD^+^ [53,54]. *C*. *difficile* is the only human pathogen to harbour this pathway, and although able to efficiently carry out NAD^+^ regeneration by Stickland metabolism, the WLP is thought to be of metabolic importance due to the up-regulation of its genes late in infection in a mouse model of CDI and its wide distribution among *C*. *difficile* strains [53,55]. The WLP locus is a 15-gene, 18.4-kb operon, whose proposed role in *C*. *difficile* is in the regeneration of NAD^+^ following a decrease in Stickland electron acceptors, which are preferentially used for NAD^+^ regeneration by so-called “acetobutyrogenesis” [25]. By coupling the acetyl-CoA/acetate generated to the production of butyrate, this was thought to regenerate NAD^+^ to sustain carbohydrate fermentation and have an increased efficiency of ATP generation [25]. This was reflected in an *acsB* mutant deficient in acetyl-CoA synthase activity, which showed a decrease in acetate and butyrate generation during in vitro growth on glucose [25]. The WLP may then allow adaptation to decreasing nutrient availability during CDI when Stickland acceptors are low, representing a means to sustain flux of carbohydrate fermentation.

### Ornithine, a key substrate in allowing *C*. *difficile* to colonise the asymptomatic gut

Asymptomatic infection of the human gut by *C*. *difficile* is thought to occur widely in populations with up to 90% of infants and 15% of adults carrying toxigenic strains [56]. Asymptomatic carriers are not only predisposed to an increased risk of infection, but also act as reservoirs of *C*. *difficile* for infection of others [57]. While there are extensive investigations of *C*. *difficile* metabolism in the gut under dysbiotic conditions [12,13,16,27,47,52], little is known about how the organism functions metabolically in asymptomatic carriers.

To date, ornithine has received most attention as a substrate of importance for the maintenance of *C*. *difficile* during asymptomatic colonisation of mice [24]. Ornithine can undergo oxidative Stickland metabolism to yield acetyl-CoA, ammonia, and alanine, involving a cascade of reactions encoded by D-ornithine aminomutase, 2-amino-4-ketopentanoate (AKP), and AKP thiolase [58]. Alternatively, it can be converted to proline via ornithine cyclodeaminase before further metabolism to 5-aminovalerate [20,24]. The importance of ornithine as a substrate in supporting growth was confirmed as deletion of genes encoding D-ornithine aminomutase *in C*. *difficile* led to a competitive disadvantage against wild-type strains during in vitro or in vivo competitive coculture [24].

Further, in an in vivo coinfection mouse model, *Clostridium sardiniese* enriched ornithine availability, leading to a significant increase in expression of putative *C*. *difficile* ornithine degradation genes allowing its import and metabolism as a Stickland substrate [10]. Furthermore, ornithine enrichment by *C*. *sardiniese* can significantly increase a putative *C*. *difficile* ornithine cyclodeaminase gene, which possibly converts it to proline for its own Stickland metabolism [10]. Conversely, upon coinfection with *P*. *bifermentans*, its generation of ornithine acts as a substrate for its own metabolism to produce proline, depriving *C*. *difficile* of an important Stickland substrate [10]. Clearly, much is still to be understood about the complex interplay between different gut commensal species and how they may promote *C*. *difficile* growth.

Mammalian macrophages act as one of the key drivers in regulating inflammation, generating either a proinflammatory, cytotoxic, type 1 inflammatory response involved in pathogen clearance, or an anti-inflammatory, tissue-repairing, type 2 inflammatory response involved in maintaining tissue integrity [59]. Ornithine is known to play a role in the immunometabolism of macrophages through the conversion of arginine to citrulline and nitric oxide, which is catalysed by nitric oxide synthase (iNOS) (*Nos2*), representative of a type 1 inflammatory response [60]. Alternatively, arginine is converted to ornithine by arginase (*Arg1*), which is representative of a type 2 inflammatory response [60].

During CDI, iNOS consumes arginine shunting it away from its conversion to ornithine by arginase [24]. A host iNOS knock-out (iNOS^−/−^) mouse infected with wild-type *C*. *difficile* shows increased ornithine concentrations in faeces, as well as an up-regulation of the *oraE* gene, suggesting its enrichment in a noninflammatory environment [24]. This was supported further in iNOS^−/−^ knock-out mice infected with the wild-type and an *oraSE* mutant, where the former showed a competitive advantage due to the availability of ornithine from ablated iNOS activity [24]. Host and diet-derived ornithine can thus be seen as an important substrate for *C*. *difficile* colonisation under noninflammatory conditions further highlighting its adaptive nature. Contrarily, CDI may then reduce ornithine levels within the gut due to the promotion of a type 1 inflammatory response, which uses arginine for iNOS production [24]. The benefit of ornithine metabolism may then be on a contextual basis, i.e., a healthy or inflamed gut environment. Therefore, the extent of ornithine’s importance during CDI warrants further investigation, such as how gut commensal species contribute towards its abundance. This would then aid our understanding of the complex relationship between the host, the gut microbiota, and *C*. *difficile* with the potential to allow for the development of a targeted therapeutic to diminish its persistence.

### Metabolism of ethanolamine by *C*. *difficile*

Once nutrients become depleted within the gut environment, the repressive effects of CcpA and CodY in *C*. *difficile* are relieved, leading to toxin production and acquisition of alternative nutrients [17,18,61]. Toxin production causes cellular damage of host epithelial cells, as well as a proinflammatory environment, leading to the liberation of phospholipids, including phosphatidylethanolamine from both microbial and host cells [62]. Phosphatidylethanolamine can then be hydrolysed to ethanolamine and glycerol through the action of diverse bacterial phosphodiesterases [63].

*C*. *difficile* can metabolise ethanolamine as a carbon and nitrogen source and occurs within a macromolecular bacterial microcompartment (BMC) [62]. This sequestration of ethanolamine metabolism is thought to protect the cell from the acetaldehyde produced and prevents its loss due to its volatile nature [64,65]. The pathway begins with the breakdown of ethanolamine into ammonia and acetaldehyde, mediated by an ethanolamine ammonia lyase protein complex, which requires the cofactor adenosylcobalamin [66]. The released ammonia can be used as a nitrogen source, whereas the acetaldehyde is metabolised to either ethanol or acetyl-CoA with the latter feeding into biosynthetic pathways or for use in substrate-level phosphorylation to form ATP [67].

The 19-gene ethanolamine (*eut*) utilisation cluster within the genome of *C*. *difficile* is induced by the presence of ethanolamine in vitro, as well as in a mouse model of CDI [10,14,24,26]. Genetic analyses has revealed that two separate polycistronic transcripts are produced spanning from *eutG*-*eutW* and *eutA*-*eutQ*, with the only form of regulation thought to be mediated by the early-sporulation sigma factor, SigF, via repression by an indirect means [26,68]. Regulation of *eut* gene clusters through the EutR regulatory protein has been reported in *Citrobacter rodentium*, enterohaemorrhagic *Escherichia coli*, and *Salmonella typhirmurium*; however, *eutR* cannot be found in the *C*. *difficile* genome [69–71]. *Enterococcus faecalis* also lacks a EutR regulator, instead using the EutV-EutW two-component system to activate the *eut* operon [72]. Ethanolamine induces these genes in *C*. *difficile* [26]; however, their regulatory activity towards the *eut* genes has not been demonstrated experimentally.

Posttranscriptional regulation of ethanolamine metabolism has been observed with a Hfq-dependent small RNA (sRNA), nc_085, repressing *eutV*, a positive regulator of a two-component system [73] and provides the first example of posttranscriptional regulation of *C*. *difficile* metabolism by a sRNA mechanism. How sRNAs regulate metabolism is an important avenue for future work as it has been noted that a number of metabolic changes occurred upon inactivation of the RNA chaperone protein, Hfq, which is involved in stabilising sRNA [74].

In a *C*. *difficile eutA* mutant, onset of pathogenesis occurred earlier than the wild type. However, ethanolamine has been shown to have no regulatory effect on known virulence factors, including toxin production, sporulation, and motility within *C*. *difficile* [26]. This observation is contrary to what has been found for other intestinal pathogens whose virulence is increased in the presence of ethanolamine [69,75,76]. Although ethanolamine has been shown to be metabolised by *C*. *difficile*, it was proposed that it is not necessary for in vivo growth but does delay the onset of pathogenesis during CDI [26]. Follow-up investigations to determine the exact regulatory mechanisms governing ethanolamine metabolism are also required to fully understand the metabolism of this important gut metabolite.

### Carbohydrates as an important source of energy for *C*. *difficile*

Carbohydrates, such as nondigestible polysaccharides in the host’s diet, are an important energy source for microorganisms residing within the large intestine. Their significance for *C*. *difficile* is evidenced by its possession of numerous genes associated with carbohydrate metabolism genes and also a raft of phosphoenolpyruvate-dependent phosphotransferase transport system (PTS) genes [13,14]. During *C*. *difficile* colonisation, these carbohydrate metabolism genes increase in expression with concomitant decreases in carbohydrate abundance due to their utilisation by the bacterium in vivo [55]. This implies that the ability of *C*. *difficile* to colonise the intestine relies on a dysbiotic microbial community lacking competitor species that would normally utilise carbohydrate sources more readily [55].

*C*. *difficile* metabolises a number of carbohydrates including, glucose, fructose, mannose, mannitol, melezitose, sorbitol, cellobiose, trehalose, and derivatives of mucin [27,28,30,31,77,78]. Carbohydrate sources are metabolised via glycolysis to pyruvate and acetyl-CoA with pyruvate converted to important intermediates for biosyntheses such as malate and oxaloacetate, lactate for NADH production or acetyl-CoA [54]. Acetyl-CoA can also be converted to biosynthetic intermediates or importantly to short chain fatty acids (SCFAs), propionate, succinate, lactate, or butyrate, regenerating NAD^+^ for further metabolism and ATP (for a full review of *C*. *difficile* central carbon metabolism, see [54]). Despite the importance of carbohydrate metabolism for *C*. *difficile*, relatively little is known about the regulation and diversity of genes associated with their utilisation save for a few molecules, which includes the sugar alcohol, sorbitol, the disaccharides, trehalose and cellobiose, and the mucosal carbohydrates, sialic acid and mannose [27,28,30–32,78].

### Host-derived sorbitol as a nutrient source during CDI

Sorbitol metabolism in *C*. *difficile* is encoded and regulated by a transcriptional antiterminator (*srlR)*, a transcriptional activator (*srlM)*, sorbitol-specific PTS components (*srlA*,*Eb* and *B*), and a sorbitol-6-phosphate dehydrogenase (*srlD*) [27]. In vitro, sorbitol induces the expression of these genes, with a *srlD* mutant unable to grow in the presence of the substrate [27]. Sorbitol is a sugar alcohol abundant in a postantibiotic environment and an in vivo mouse study reported its utilisation during the early stages of colonisation in CDI [12,55]. Separately, a toxin-deficient *C*. *difficile* mutant had lower expression of its sorbitol metabolism genes in vivo in comparison to the wild type, suggesting the metabolism of sorbitol following toxin-mediated inflammation [27]. Sorbitol has also been shown to be consumed in vivo, with the up-regulation of aldose reductase (which converts glucose to sorbitol) by immune cells and host epithelial cells during toxin-mediated inflammation [27]. Additionally, the utilisation of sorbitol lowers *C*. *difficile* toxin production in vitro and in vivo, while a *srlD* mutant increases its production in vivo [27]. This suggests that the absence of sorbitol metabolism augments toxin production to increase inflammation [27]. Subsequently, host aldose reductase would be up-regulated by toxin-mediated inflammation allowing for the acquisition of sorbitol by *C*. *difficile* [27]. Finally, similar to above, sorbitol metabolism genes were shown to be up-regulated in vivo in streptomycin-treated mice infected with *C*. *difficile* [13]. Altogether, toxin-mediated inflammation plays a role in acquiring sorbitol for a nutrient source in a diet-independent manner. Following inflammation, immune cells and host tissue express aldose reductase, producing sorbitol, which is then released following damage by toxin activity to then be utilised by *C*. *difficile*.

### Trehalose utilisation and hypervirulence: Are they linked?

Trehalose is a disaccharide consisting of two glucose molecules bonded together by an α 1,1 glycosidic bond, which can be used by microorganisms not only as a carbon and energy source but also as an osmoprotectant [79,80]. Compared with other bacterial species *C*. *difficile* has a heterogeneous arrangement of the typical trehalose operon, where strains belonging to ribotypes (RT) 012, 027, 017, and 078 possess the canonical *treR* and *treA* genes encoding a trehalose regulatory protein and phosphotrehalase, respectively, with no equivalent of a *treP* (which encodes a trehalose-specific PTS for transport of trehalose into the cell) [28,29,81]. In addition, some strains belonging to RT023 and 078 possess a secondary cluster of genes associated with trehalose metabolism, which includes the orthologues *treA2* and *treR2*, the putative trehalase gene *treX* (which is truncated in RT023 strains), and the trehalose-specific PTS *ptsT* [28,29].

**Fig 1:**
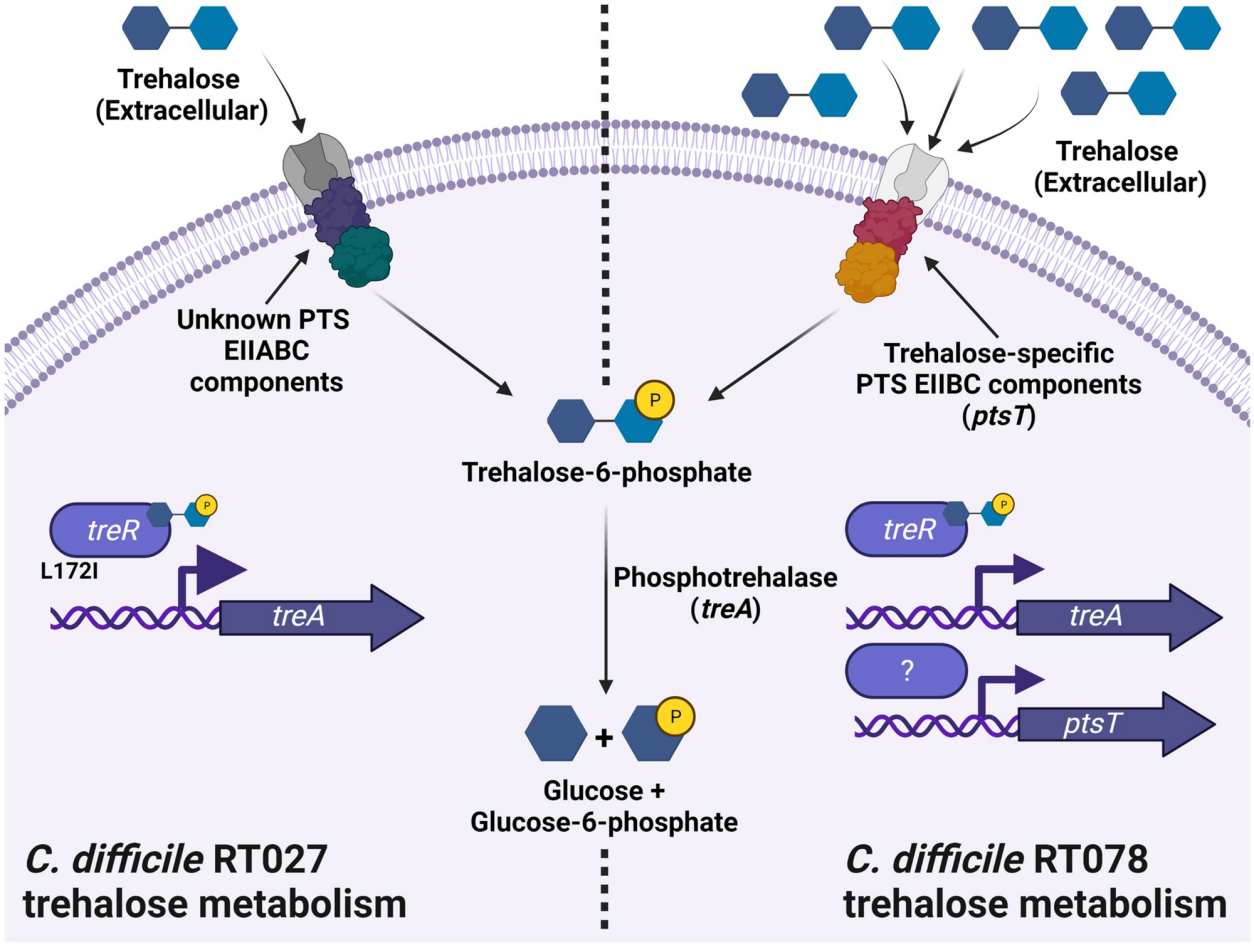
Schematic diagram of trehalose metabolism in the *C*. *difficile* ribotype (RT) 027 and 078 lineages. The *C*. *difficile* RT027 trehalose metabolism is thought to occur with the transport of trehalose into the cell by an unknown phosphotransferase system (PTS) transporter, which is concomitantly phosphorylated to trehalose-6-phosphate (T6P) during uptake. The TreR regulatory protein then binds T6P leading to derepression of the *treA* gene producing the phosphotrehalase enzyme. Due to the proposed sensitivity of the TreR regulatory protein to low concentrations of trehalose-6-phosphate, this is thought to allow increased *treA* gene expression levels. The phosphotrehalase enzyme degrades T6P to glucose and glucose-6-phosphate, which enter glycolysis and promote growth. The *C*. *difficile* RT078 trehalose metabolism is thought to occur with the transport of trehalose into the cell by the trehalose-specific PTS transporter, encoded by the *ptsT* gene, which is concomitantly phosphorylated to T6P during uptake. The rapid acquisition of T6P into the cell is thought to then interact with the TreR regulatory protein leading to the induction of the *treA* gene as described above, as well as probably inducing the expression of the *ptsT* gene to allow further uptake of trehalose. Molecular structures acquired from Chemspider.com. Created with Biorender.com.

Recently, strains from RT027 and RT078 were shown to be capable of growth in vitro on trehalose (10 mM) at concentrations much lower than required by other *C*. *difficile* isolates (50 mM) [28]. It has been proposed that an increased sensitivity to trehalose in RT027 occurs due to a L172I amino acid substitution in the TreR protein [62]. To support this, when non-RT027 strains produced spontaneous mutations in their *treR* gene, they were also capable of utilising trehalose when present at 10 mM [28]. It is proposed that mutations within TreR allow increased sensitivity to low concentrations of trehalose and in turn lead to increased expression of the *treA* gene [28] (Fig 1). As with RT027, RT017 strains also possess a TreR mutation with a C171S amino acid substitution that also allows for growth and expression of the *treA* in low concentrations of trehalose [78].

In RT078 strains, the secondary cluster of genes encoding trehalose metabolism are thought to be responsible for their ability to metabolise low concentrations of trehalose. Specifically, through a series of elegant experiments, *ptsT* was shown to be essential for this phenotype (Fig 1) [28].

Intriguingly, a trehalose-enriched diet is linked to increased disease severity in mice, seemingly associated with elevated levels of TcdB, when infected with the hypervirulent R20291 strain (RT027) [28]. In addition, *treA* gene expression is up-regulated, along with other carbohydrate hydrolase genes, in clindamycin-treated mice [13]. Taken together, these observations would appear to implicate trehalose, and its metabolism by *C*. *difficile*, with hypervirulence. More recently, however, in a different mouse model of CDI using a RT027 strain, a trehalose-enhanced diet led to no increase in mortality and surprisingly lowered *treA* gene expression and also TcdA and TcdB loads [82]. Importantly, there were significant methodological differences in these two studies with Collins and colleagues (2018) supplying mice with trehalose postcolonisation of *C*. *difficile*, which was replenished daily, while Zhang and colleagues (2020) supplied trehalose precolonisation of *C*. *difficile* in a single feed [28,82]. Subsequently, in a report of an in vitro bioreactor model of CDI using again a RT027 strain, it was concluded that the presence of trehalose in a postantibiotic environment does not stimulate CDI, as increased competition for trehalose by remaining members of the microbial community allowed only for *C*. *difficile* spore germination while preventing toxin production [83].

Interest in the distribution of the variant TreR across *C*. *difficile* clade 2 and 4 isolates found its occurrence to be more frequent than previously thought and was actually clade specific rather than ribotype specific [84]. Additionally, the secondary gene cluster of trehalose metabolism genes found in RT078 strains could be found in isolates across all five *C*. *difficile* clades, and, importantly, its presence lacked any association with increased mortality in CDI cases [84,85]. It can be concluded that although trehalose metabolism variants are present throughout the *C*. *difficile* species, these genotypes were established long before the widespread introduction of synthetic trehalose into the human diet since 2000. Indeed, the hypothesis that *C*. *difficile* exhibited increased virulence due to this increase in trehalose in the human diet is disputed, as it is not supported by CDI patient data nor human gut models.

### The role of cellobiose in *C*. *difficile* pathogenicity

Cellulose, a complex carbohydrate found in plant-based foods in the human diet, acts as a major nutrient source for the colonic microbiota [86]. Cellulose degradation produces the disaccharide cellobiose, which can be metabolised by *C*. *difficile* by an operon containing a cellobiose-specific PTS (*celABC*), a 6-phospho-beta glucosidase (*celF*) and a repressor regulatory protein *(celR*) [30].

Knock-out experiments have shown *celA* to be essential for growth on cellobiose [30]. CelR was shown to induce transcription from the cellobiose metabolism operon with cellobiose-6-phosphate likely the inducer molecule resulting in CelR binding upstream of *celA* [30]. CelR also possesses autoregulatory activity for its own transcription with neither it nor the *cel* operon under control of carbon catabolite repression (CCR) [30].

Interestingly, uptake of cellobiose in *C*. *difficile* and CelR, in particular, was found to have an impact on sporulation and toxin production [30]. It was proposed that a distally transcribed phosphodiesterase gene under control of CelR had a function to degrade the signalling molecule c-di-GMP, which, in *C*. *difficile*, is involved in negatively regulating toxin production and sporulation [87–89]. Therefore, the inability to repress this phosphodiesterase gene by CelR leads to a lower level of c-di-GMP allowing increased toxin and sporulation rates [30]. Finally, in a golden Syrian hamster model, a *celA* mutant showed lowered levels of colonisation, as well as the inability to cause recurrent infection [30]. These findings suggest that the cellobiose operon plays a role in *C*. *difficile* pathogenesis by affecting the rate of sporulation and toxin production. Altogether, cellobiose utilisation and its associated regulatory processes are shown to play an important part in *C*. *difficile* pathogenesis. Nutrient-specific metabolic regulators in *C*. *difficile* then may have broader roles in its physiology by indirectly modulating aspects of virulence. This then should encourage the identification of nutrient-specific regulator target genes outside of their canonical metabolic operons.

### Microbiota-liberated mucous degradation products support *C*. *difficile* growth

Mucous found on the gut lining of the large intestine consists of mucin, a glycosylated protein that contains ribose, N-acetylglucosamine, fucose, mannose, and sialic acid [90]. These can act as a major carbohydrate source for the gut microbiota [91,92]. In vivo mouse models of CDI have highlighted the importance of sialic acids such as 5-acetylneuraminate as an important nutrient to support *C*. *difficile* colonisation during infection [12,55]. In a gnotobiotic mouse model, *Bacteroides thetaiotaomicron* liberates free sialic acid from mucin to support the growth of *C*. *difficile* [31]. While *C*. *difficile* lacks the genes required to cleave sialic acid from host mucin, it does possess the *nan* operon encoding the catabolic pathway for sialic acid [11], with increased expression being observed during co-colonisation of *B*. *thetaiotaomicron* and *C*. *difficile* in gnotobiotic mice [31]. Sialic acid utilisation by *C*. *difficile* in vivo was further highlighted in a conventional mouse model following antibiotic use, which showed increased sialic acid levels with an associated up-regulation of the *C*. *difficile nan* operon postinfection [31]. This suggests that disruption of the gut microbiota allows *C*. *difficile* to exploit the now readily available nutrients that would otherwise be efficiently consumed by other bacterial species in an unperturbed environment. Similar phenomena have been observed during *Salmonella typhimurium* infection, whereby gut microbiota metabolites, including sialic acid, influence pathogen colonisation [31,75,93].

In a similar study, the mucin-degrading commensal microorganisms, *Akkermansia muciniphila*, *Ruminococcus torques*, and again *B*. *thetaiotaomicron*, have been shown to facilitate the growth of *C*. *difficile* by cross feeding when mucin is the sole carbon source during coculture and from cell-free supernatant filtrates [32]. Specifically, the mucin monosaccharide mannose acts as a chemoattractant for *C*. *difficile*, which supports increased growth relative to glucose [32]. Mannose utilisation genes are thought to have undergone positive selection in a number of *C*. *difficile* ribotypes, where its use as a sole carbon source facilitates robust growth in several *C*. *difficile* strains [28,94,95]. This demonstrates the conserved ability to utilise mucin-related sugars across the *C*. *difficile* species and, thus, their importance in its lifestyle. Similar findings of cross feeding from mucin to facilitate pathogen growth have also been shown during the coculture of vancomycin-resistant *Enterococcus* and *R*. *torques*, with the former unable to grow in mucin alone [96]. Mucin degradation by commensal microorganisms may then play a key role in *C*. *difficile* pathogenesis, whereby the release of monosaccharide by-products is thought to facilitate colonisation of the intestinal mucous layer by chemotaxis while also providing substrates for growth. It is then plausible there is a level of synergy between *C*. *difficile* and mucin-degrading commensal microorganisms in the perturbed gut environment; therefore, deeper insight into whether other gut community members support CDI progression are warranted to fully understand how this pathogen colonises the gut.

### Zinc limitation impacts *C*. *difficile* metabolic adaptation

Micronutrients play an essential role in many cellular processes in microorganisms and are considered to be under constant limiting amounts within the gut environment. Pathogens can acquire micronutrients through the release of metal chelating molecules, the use of high-affinity metal transporters, or the exploitation of the metal scavenging mechanisms of other microorganisms to acquire divalent metal ions such as iron, zinc, manganese, and cobalt [97].

Zinc (Zn) is an important micronutrient that plays an essential role in the structural and functional activity of many proteins [97]. In *C*. *difficile*, Zn limitation caused by the metal-chelating protein calprotectin in vitro leads to the expression of the *zupT* gene, predicted to have a role as a transporter of a diverse range of metal ions [33,46]. ZupT is essential for zinc uptake, and its disruption is detrimental to *C*. *difficile* growth, colonisation, and recurrence in an in vivo mouse model of CDI [33]. In addition, calprotectin leads to the increased expression of the *prdA* gene during Zn sequestration; however, during this, in vitro, the PrdB subunit of PR requires selenium for its catalytic activity [22,46]. Selenium levels were also reduced within a mouse model of CDI in vivo; therefore, unless selenium is supplemented, proline reductase activity may be limited during calprotectin-mediated Zn limitation [46]. Further, the inability to utilise selenium due to disruption of *selD*, which encodes the selenophosphate synthetase, is thought to lead to a shift in metabolism from Stickland fermentation of proline to mannitol fermentation, as mannitol utilisation genes are induced within this *selD* mutant [98]. Mannitol is a sugar alcohol that shows increased abundance in a postantibiotic environment, where following *C*. *difficile* colonisation, it decreases in abundance over time [12,55]. In addition, in a cefoperazone-treated mouse model of CDI, mannitol catabolism genes were shown to be up-regulated in *C*. *difficile*, and it can be used as a primary nutrient source in minimal medium [12,13]. Given the dependence of selenium to allow PR activity, *C*. *difficile* may then adapt to this limitation by utilising mannitol to potentially regenerate NAD^+^ [99] to sustain colonisation. The *C*. *difficile* metabolic response to calprotectin may then be influenced by the dynamic nutrient landscape found within the host, which drives its growth, specifically regulating proline reduction in combination with the abundance of selenium, which is required for this process [22].

Lopez and colleagues (2019) then proposed that the induction of proline reductase by Zn limitation was part of a broader metabolic adaptation in which the effects of calprotectin indicate an immune response and, in turn, inflammation mediated by *C*. *difficile* toxin activity [46]. As mentioned above, toxin-mediated inflammation induces the degradation of collagen-producing hydroxyproline and proline, which, in this context, could explain the induction of proline reductase in the inflamed gut [16,23]. This is further supported through the induction of the *eut* genes by calprotectin, involved in ethanolamine metabolism, which are increased during toxin-mediated inflammation as described above [26,46]. Therefore, calprotectin may benefit *C*. *difficile* as it inadvertently acts as one signal to indicate an inflamed gut, leading to metabolic adaptation so it can capitalise on those nutrients now available.

### *Para*-cresol as a bacteriostatic metabolic by-product of *C*. *difficile*

*C*. *difficile* can metabolise tyrosine to an intermediate, *p*-hydroxyphenylactate (*p*-HPA), and, subsequently, to *p*-cresol [34], a bacteriostatic phenolic compound that inhibits growth of gut microbiota species, including *Escherichia coli*, *Klebsiella oxytoca*, and *Proteus mirabilis* [100]. *p*-Cresol is thought to increase membrane permeability and hence the loss of small molecular compounds such as phosphate from other bacteria [100]. Importantly, *C*. *difficile* has a tolerance towards *p*-cresol, although this varies from strain to strain and its importance in colonisation and/or pathogenesis remains unclear [34].

HpdBCA decarboxylase, encoded by the *hpdBCA* operon, found across all 5 toxigenic *C*. *difficile* clades, is required for the conversion of *p*-HPA to *p*-cresol [34,101,102]. As would be expected, the production of this bacteriostatic compound provides a distinct advantage on *C*. *difficile* as observed during coculture experiments with gut commensals in vitro [100]. The importance of *p*-cresol for sustaining infection was further exemplified by the fitness defect of a *C*. *difficile hpdC* mutant in an in vivo mouse relapse model of CDI [100]. The *hpdBCA* operon is induced by *p*-HPA rather than tyrosine [35], and, interestingly, sources of *p*-HPA within the gut in addition to *C*. *difficile*’s own biosynthetic capability are thought to include being derived from host cells, and other gut commensals including *Klebsiella* and other *Clostridium* species [103].

Upstream of the *hpdBCA* operon, a SigA consensus sequence, has been found, which facilitates induction in the presence of *p*-HPA even if provided exogenously [35]. However, at the time of writing, the identity of the protein that regulates this operon and the transporter, allowing *p*-HPA uptake, has yet to be identified [35]. Interestingly, unlike the variation in tolerance towards *p*-cresol, all strains so far examined show similar levels of efficiency of *p*-HPA conversion to *p-*cresol, with production being 30-fold higher in the presence of *p-*HPA than compared to tyrosine supplementation [102]. Despite the absence of a specific regulatory protein for the *hpdBCA* operon, CodY, the nutrient-sensing global regulator, has been shown to be indirectly involved in its regulation [102]. This may occur as putative tyrosine ABC transporter genes, CDR20291_0805 and CDR20291_0806, in *C*. *difficile* R20291 strain were shown to contain CodY consensus binding sequences in their promoter regions [104,105]. Finally, the presence of *p*-HPA in excess amounts was shown to be inhibitory to *C*. *difficile* growth; in turn, HpdBCA activity provides a means to lower *p*-HPA concentrations in the gut environment to a tolerable level [102]. Altogether, it can be seen that the production of *p*-cresol as a by-product of *p-*HPA, and possibly tyrosine, metabolism governs *C*. *difficile*’s success within the gut environment by diminishing the numbers of gut commensal species that establish colonisation resistance towards it.

*p*-Cresol production is one factor that prevents the reestablishment of antagonistic gut microbiota species to sustain dysbiosis and, thus, *C*. *difficile* colonisation; however, as a whole, these mechanisms are poorly understood. Understanding the processes *C*. *difficile* uses to suppress growth of the gut microbiota is critical in order to fully understand its pathogenesis. Therefore, given the importance of the gut microbiota in preventing *C*. *difficile* colonisation, inhibiting these processes could act as an effective treatment to restore antagonistic species towards *C*. *difficile*, in turn reestablishing gut homeostasis.

## Conclusions

**Fig 2:**
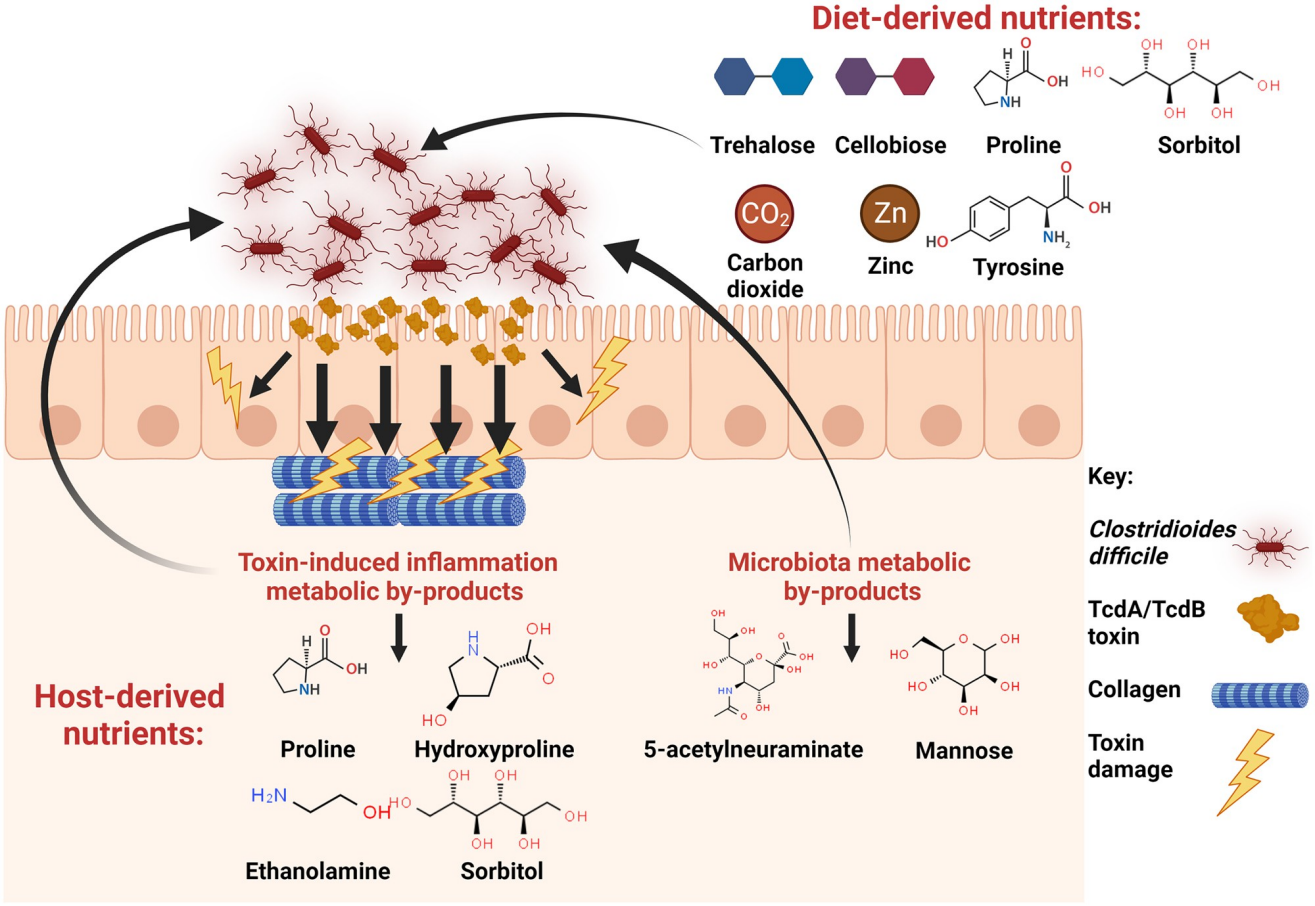
Overview of the nutrients *C*. *difficile* utilises and their origin during infection of the gut. Molecular structures acquired from Chemspider.com. Created with Biorender.com.

*C*. *difficile*’s ability to capitalise on a wide array of nutrients classifies it as a bacterial generalist for nutrient acquisition, possessing high genetic flexibility to metabolise a range of nutrients from its environment. The ability to effectively metabolise nutrients available in the gut environment is the key to the success of a pathogen colonising and establishing infection, where in *C*. *difficile*, a number of nutrient sources are shown to be important for survival and persistence (Fig 2). Host and diet-derived nutrients, such as amino acids in the form of proline and hydroxyproline, are the primary means of NAD^+^ regeneration to drive catabolic flux in metabolism, being highlighted several times as important nutrients during *C*. *difficile* colonisation [4,16,20,23,47]. During limitation of these Stickland substrates, *C*. *difficile* may utilise the WLP as an adaptive response to maintain redox balance, thus sustaining the catabolic flux of carbohydrate fermentation [25]. The use of diet and host-derived ornithine represent the first instance of describing *C*. *difficile* metabolism under homeostatic conditions, specifically the production of ornithine by immunometabolism, which is thought to contribute to persistence within its host [24]. Carbohydrates and ethanolamine act as important sources of energy, with carbohydrates thought to play a role in some aspects of virulence during colonisation and infection [26–28,30–32,78]. Metal chelation by the immune calprotectin protein can be thought to signify an inflamed gut to *C*. *difficile*, rewarding it with an appropriate metabolic adaptation to thrive on metabolites abundant in this environment such as proline, hydroxyproline, and ethanolamine [16,46]. Production of *p*-cresol is an important metabolic trait in *C*. *difficile*, as it provides a competitive advantage over other gut commensal species paving the way for continued dysbiosis [34,35,100,102].

A hallmark of *C*. *difficile* metabolism studies are their reliance on the ability to produce mutant strains to provide evidence of a gene’s function. Further work using the ClosTron mutagenesis [106], allelic exchange system [107], and CRISPR-Cas9 [108] genetic tools will further decipher and unlock the roles of these gene products to better solidify *C*. *difficile’*s metabolic capabilities in the context of pathogenesis. The understanding of aspects of *C*. *difficile*’s metabolism, which aid in its success as a pathogen, is slowly being realised, and continued research will further elucidate the adaptive mechanisms that contribute towards *C*. *difficile*’s success during infection.

Understanding the metabolic needs of *C*. *difficile* also has the potential to provide an insight into those gut microbiota species that may compete or efficiently consume its preferred metabolites in the gut. The use of gut competitor species as probiotic therapeutics then represents a novel strategy for the treatment of *C*. *difficile* infection; however, whether these can achieve clinical efficacy remains unanswered. Theoretically, probiotic therapeutics could reestablish colonisation resistance in the gut through the exclusion and consumption of those nutrient sources that are integral to *C*. *difficile* success and pathogenesis. Much is still to be learned about the metabolic aspects of CDI and how this shapes the pathogenesis of this important gut pathogen.
